# Sheehan Syndrome Presenting with Psychotic Manifestations Mimicking Schizophrenia in a Young Female: A Case Report and Review of the Literature

**DOI:** 10.1155/2020/8840938

**Published:** 2020-12-04

**Authors:** Nipun Lakshitha de Silva, Janith Galhenage, Madhubhashinee Dayabandara, Noel Somasundaram

**Affiliations:** ^1^Diabetes and Endocrine Unit, National Hospital of Sri Lanka, Colombo 10, Sri Lanka; ^2^University Psychiatry Unit, National Hospital of Sri Lanka, Colombo 10, Sri Lanka

## Abstract

**Introduction:**

Sheehan syndrome presents with features of multiple hormone deficiencies including lactation failure and amenorrhoea as well as with features of central hypothyroidism and adrenocorticotropic hormone deficiency. Psychiatric manifestations are mostly limited to cognitive impairment. Psychotic presentations are rare and limited to case reports. *Case Presentation*. A 32-year-old female was evaluated for fearfulness and delusions for one year. She had persecutory and bizarre delusions, delusion of thought possession, and elementary auditory hallucinations. These began four months after the birth of her third child. The delivery had been complicated with postpartum haemorrhage. Her symptoms caused the functional decline and progressively worsened, resulting in suicidal ideation. Cognitive assessment revealed mild impairment in attention. Further inquiry revealed lethargy, constipation, cold intolerance, and lactation failure. She was slow, having dry skin, puffy face, and bradycardia with a blood pressure of 80/60 mmHg (supine) and 70/50 mmHg (standing). She had hyponatraemia, elevated creatine phosphokinase, low thyroxine, prolactin, FSH, LH, and IGF-1. She had poor cortisol and growth hormone response to the insulin tolerance test. MRI-pituitary showed empty sella. A diagnosis of Sheehan syndrome was made. Her symptoms improved completely after the initiation of levothyroxine and hydrocortisone.

**Conclusions:**

Sheehan syndrome can present with psychotic symptoms mimicking schizophrenia with variable involvement of cognition. Detailed reporting of these patients would enhance better characterization of the clinical presentation and risk profile of these patients.

## 1. Introduction

Sheehan syndrome is postpartum hypopituitarism caused by necrosis of the pituitary gland. This is thought to be a result of hypotension caused by postpartum haemorrhage. The risk is higher in those with predisposing factors of the enlarged pituitary gland during pregnancy, small sella turcica, vasospasm, and a thrombophilic state [1]. The prevalence of Sheehan syndrome was high in an era of poor obstetric care. There has been a marked reduction due to improved obstetric care especially in the developed world.

The presentation of Sheehan syndrome can vary; some patients are diagnosed soon after childbirth while delayed diagnosis even up to 20 years has been reported [1, 2]. Common manifestations include failure of lactation, amenorrhoea, and asthenia [2–4]. Psychiatric manifestations are less common and are mostly limited to cognitive impairment [2]. Psychotic manifestations with preserved cognitive functions are limited to few case reports. We report a patient who had Sheehan syndrome presenting one year after delivery with psychotic symptoms that were similar to schizophrenia.

## 2. Case Presentation

A 32-year-old Sri Lankan female presented to the psychiatry services with fearfulness, undue suspicion, and hearing voices. She believed that somebody has implanted a camera inside her head to record her thoughts. She was irritable. In addition, she had begun to neglect her functions as a housewife and a mother. These symptoms were present for one year and had worsened over the previous two weeks.

Over the previous year, she was also experiencing lethargy, constipation, and cold intolerance. Symptom review was negative for postural dizziness, polyuria, polydipsia, headache, and visual disturbances.

She was a mother of three children; the youngest was one year and four months old. Her last pregnancy was complicated with gestational diabetes and pregnancy-induced hypertension. Childbirth by vaginal delivery was complicated with heavy bleeding. She experienced inability to lactate her child. An intrauterine contraceptive device was inserted six weeks postpartum. She did not experience any menstrual bleeding since childbirth.

She had no other comorbidities. Assessment of personality indicated that she was a well-adjusted person prior to the onset of illness without any maladaptive traits. This was her first presentation to a psychiatry service.

She was from a poor socioeconomic setting having poor family support, and her husband was alcohol dependent. This has led to a delay in seeking healthcare advice.

She was average built with a weight of 49 kg and a height of 151 cm. She was slow in response with dry skin of normal complexion and facial puffiness. There was no goitre. She had bradycardia (heart rate of 56/min) with a supine blood pressure of 80/60 mmHg and a standing blood pressure of 70/50 mmHg. The rest of the physical examination was normal.

Her mental state examination revealed an anxious mood with fleeting passive suicidal ideas. She had persecutory delusions involving an unidentified persecutor and a bizarre delusion that an external object has been implanted in her head to monitor her mental activity. She also had delusions of thought possession and elementary auditory hallucinations. Her delusions and hallucinations were observed to be transient. They were changing throughout the hospital stay. Initial persecutory delusions disappeared and the content of bizarre delusions changed in subsequent mental state examinations.

Her orientation with regard to time, day, month, place, and city was normal. Attention and concentration tested with digit span were impaired, and she could repeat only four digits forward (normal is 5 to 9 digits) and three in reverse (normal being 6 or more). Her short-term and long-term memory as well as other aspects of extended cognitive assessment was normal.

Initial laboratory investigations showed hyponatraemia, elevated creatine phosphokinase, and low free T4 with normal thyroid-stimulating hormone (TSH) (Table 1). A provisional diagnosis of organic delusional disorder (schizophrenia-like) was made and risperidone 1 mg daily was started.

**Figure 1:**
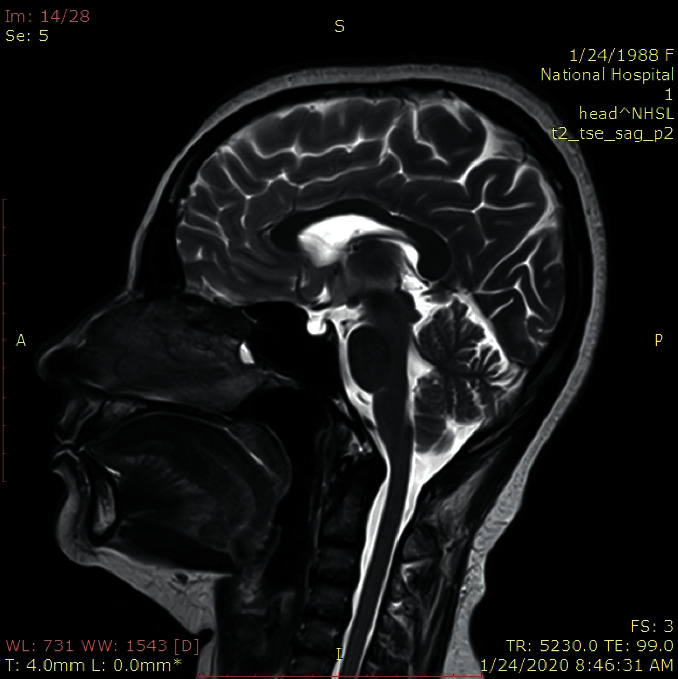
MRI-pituitary of the patient showing empty sella. T2 sagittal section of MRI-sellar region shows normal-sized pituitary fossa filled with CSF. The pituitary gland is not seen in the sella.

She was referred to the endocrinology team for further evaluation and management. With a 9am cortisol of 221 nmol/L, she was started on levothyroxine under the cover of hydrocortisone. After normalizing free T4, she underwent an insulin tolerance test withholding hydrocortisone for 24 hours (Table 1). A diagnosis of panhypopituitarism was made. MRI of the pituitary gland showed a normal-sized sella turcica filled with cerebrospinal fluid (CSF). There was no pituitary gland, suggesting empty sella. The pituitary stalk was slightly deviated to the right extending up to the sellar floor showing normal signal and diameter (Figure 1).

Levothyroxine and hydrocortisone were continued. Symptoms, including psychotic symptoms and hyponatraemia, improved remarkably within two weeks and risperidone was discontinued. Oestrogen and progesterone replacement therapy was initiated later. The patient is currently satisfied with her clinical outcome.

## 3. Discussion

Our patient presented to a psychiatry service with disturbing delusions and hallucinations with fairly intact cognition. The presentation would usually fit into a schizophrenia-like disorder.

However, detailed history and clinical examination suggested an organic pathology with the presence of hypothyroid features. Early endocrine referral led to the confirmation of diagnosis and initiation of specific treatment leading to marked clinical improvement. The diagnosis of Sheehan syndrome was made based on the temporal relationship of symptoms to childbirth, history suggestive of postpartum haemorrhage, characteristic anterior pituitary dysfunction, and imaging evidence of empty sella. Low prolactin leading to lactation failure noted in our patient is characteristically seen in Sheehan syndrome. Most of the other causes of hypopituitarism including lymphocytic hypophysitis cause normal or elevated prolactin [1]. This further strengthened our clinical diagnosis.

The delusions of thought possession and the bizarre delusion initially suggested the possibility of schizophrenia. Organic psychosis can present with persecutory delusions and even first rank symptoms or thought disorder. Patients with intact cognitive function are known to present with complex delusions such as in this case. A strong suspicion of organic psychosis was aroused due to the changing nature of her symptoms coupled with physical signs [5].

Psychotic manifestations in patients with Sheehan syndrome are limited to several case reports. We performed a systematic literature search in PubMed and Google Scholar to identify case reports on psychotic manifestations in Sheehan syndrome. Further reports were recognized using cross-referencing of the initial articles. Articles which did not provide full text in English were excluded. Thirteen full-text articles were reviewed for eligibility. One reported a patient without clear psychotic manifestations [6]. Another report described a patient with delirium precipitated with sepsis and severe hyponatraemia. There were no psychiatric manifestations until the acute insult [7]. Another patient misdiagnosed with depressive disorder did not have any psychotic manifestations [8]. Though one report described protracted delirium as a result of Sheehan syndrome, the original presentation was with seizures and cerebral oedema associated with hyponatraemia and hypoglycaemia [9].

After excluding the above cases, nine reports of women with Sheehan syndrome presenting with psychotic manifestations were reviewed in detail [10–18]. Clinical details of these patients are summarised in Table 2. In one patient, details of lactation were not reported. All the other patients had evidence of lactation failure and secondary amenorrhoea. Secondary hypothyroidism and ACTH deficiency were noted in all the patients. None had diabetes insipidus. Growth hormone deficiency was not tested in any of the patients. All patients except one patient had a clear history of postpartum haemorrhage. Except the first three patients reported before 1980, all the other patients had imaging evidence of empty sella. None of the patients had prior psychiatric comorbidities.

The interval between childbirth and onset of psychiatric manifestations ranged from few days [10, 14, 15] to about three decades. There seems to be a bimodal distribution. Few patients present acutely and others develop psychiatric symptoms more than few years later. In patients who developed psychiatric manifestations later, most had developed lactation failure and amenorrhoea soon after childbirth, suggesting early-onset hypopituitarism. But the time of the onset of hypothyroidism and ACTH deficiency was not clear from the reports in some [16–18]. In two patients, other features of hypopituitarism have lasted long before the onset of psychiatric manifestations [11, 12]. In our patient, symptoms have developed early though they were left unattended.

Our patient had psychotic manifestations with mildly impaired attention and concentration but otherwise intact cognitive functions. Cognitive involvement, attention, and concentration were variable among different patients in the reported cases. Details of these aspects were not reported in five patients. Of the other four, one had normal cognition, attention, and concentration [13]. One had impaired cognition and disorientation [11]. Two patients had intact memory with impaired attention and concentration [15, 18]. This is in contrast to the observation that impaired cognition is one of the commonest manifestations of Sheehan syndrome [2].

The pathophysiology of psychiatric manifestations in Sheehan syndrome is not clear. Psychiatric symptoms are hypothesized to be caused by the complex interaction of hormonal deficiencies with metabolic and electrolyte changes in the central nervous system [19]. All patients have had dramatic improvement with glucocorticoid and thyroxine replacement therapy, suggesting a possible link to these hormonal deficiencies. None of the patients received growth hormone and oestrogen/progesterone replacement therapy. Therefore, growth hormone and gonadotropin deficiencies seem to be of lesser clinical relevance. Hyponatraemia is also known to cause psychiatric manifestations. However, it is usually associated with delirium presenting with subtle cognitive deficits to overt fleeting psychotic symptoms with global impairment of consciousness [20]. Frank, well-formed delusions and hallucinations in the absence of impairment of consciousness and neurological signs have not been reported in hyponatraemia [19, 21, 22]. Hypothyroidism may be more important in pathogenesis as it is consistently associated with neuropsychiatric manifestations [23]. Hypothyroidism is associated with mood symptoms rather than psychosis though overt psychotic presentations without significant mood symptoms have been reported [24].

Psychosis has been noted in male and female patients with hypopituitarism due to other causes [25–29]. Therefore, it could be a rare manifestation of hypopituitarism per se. The postpartum state is known to predispose to psychosis. Lactation failure characteristically seen in Sheehan syndrome might have a psychological impact on the woman. At least in patients developing psychiatric manifestations early, these might be contributory. Postpartum psychosis typically presents within the first 3–10 days after childbirth [30]. It is noted for its delirium-like presentation with rapid mood fluctuations.

There are several unanswered questions regarding psychotic manifestations in patients with Sheehan syndrome. Long interval between symptoms and onset of hormone deficiencies in some patients remains elusive. Whether there are any predisposing factors for psychiatric manifestations in these patients compared to a large number of patients with Sheehan syndrome without any psychotic manifestations is another interesting question. Detailed attention to patients' personality traits and triggering external factors might help to find answers.

## 4. Conclusions

This rare presentation of Sheehan syndrome with psychosis not only represents the close association of organic pathology to psychiatric manifestations but also illustrates the possible psychiatric adverse effects of panhypopituitarism and its metabolic consequences.

## Figures and Tables

**Table 1:** Summary of laboratory investigations.

Investigation	Result	Reference range
Haemoglobin (g/dL)	11.8	11–16

Serum creatinine (mg/dL)	0.84	0.5–1.1

Sodium (mmol/L)	122	135–145

Potassium (mmol/L)	3.8	3.5–5.1

Aspartate transaminase (U/L)	56	<40

Alanine transaminase (U/L)	22	<40

Creatine phosphokinase (U/L)	1381	26–192

Fasting blood glucose (mg/dL)	96	<100

Thyroid-stimulating hormone (mIU/l)	3.428	0.5–4.7

Free T4 (ng/dl)	<0.1	0.89–1.76

9am cortisol (nmol/l)	221	118–618

Prolactin (mIU/l)	26.94	59–619

Follicle-stimulating hormone (IU/L)	2.88	

Luteinizing hormone (IU/L)	0.94	

Insulin-like growth factor (ng/ml)	8	177–382

Insulin tolerance test		
Lowest random blood glucose (mg/dL)	13.9	<40
Highest growth hormone (*μ*g/L)	<0.1	>7
Highest cortisol (nmol/L)	88.1	>550

**Table 2:** Summary of case reports describing patients presenting with psychotic manifestations due to Sheehan syndrome.

Author, year	Age at presentation	Interval between psychiatric manifestations and childbirth	Psychiatric manifestations	Hormone deficiencies^*∗*^	Notes
Lightenberg and Cader, 1959 [10]	37 years	12 days	Auditory and olfactory hallucinations, delusions. Apathy, disorientation. No details on other cognitive functions.	TSH, ACTH, gonadotropin, and prolactin	Twice improves without replacing deficient hormones, but relapsed. MRI-pituitary not reported.

Hanna, 1970 [11]	55 years	19 years	Delusions and auditory hallucinations Impaired cognition including disorientation and poor memory.	Gonadotropin, TSH, ACTH. Symptoms of hypopituitarism have lasted long before psychiatric manifestations No details about prolactin levels or lactation	One week after presentation developed seizures, coma, and hypotension. MRI pituitary not reported.

Kitis and Johnson, 1976 [12]	48 years	10 years	Auditory hallucinations, paranoid delusions, and aggressive behavior. Depression. No details on cognition, attention, and concentration.	Gonadotropins, prolactin, TSH, ACTH (hormone deficiency persisted at least 4 years before the onset of psychiatric manifestations)	Six years after childbirth, Sheehan syndrome diagnosed, but the patient defaulted. MRI pituitary not reported.

Leo et al., 1998 [13]	57 years	29 years	Paranoid delusions and auditory hallucinations. Apathy and dysphoria. Normal memory and attention	Gonadotropins, prolactin, TSH, ACTH (onset of any of the hormone deficiencies not clear from history)	Initially diagnosed as schizoaffective disorder with poor response.

Kale et al., 1999 [14]	21 years	15–20 days	Hallucinations, persecutory delusions, and delusions of infidelity.Orientation normal. No cognitive assessment.	Gonadotropins, prolactin, TSH, ACTH	No history of postpartum haemorrhage.

Shoib, 2013 [15]	31 years	16–18 days	Persecutory delusions, auditory hallucinations. Intact memory, poor attention, concentration, and verbal fluency.	Gonadotropins, prolactin, TSH, ACTH	

Reddy, 2017 [16]	42 years	11 years	Delusions of persecutions and second person auditory hallucinations. Has forgetfulness, no details on cognition, attention, and concentration.	Gonadotropins, prolactin (onset soon after childbirth) TSH, ACTH (onset not defined)	

Nath et al., 2018 [17]	43 years	13 years	Catatonia, reduced speaking, and reduced emotional reactivity. No details about cognition, attention, and concentration.	Gonadotropins, prolactin (onset soon after childbirth) TSH, ACTH (onset not defined)	

Shiekh et al., 2018 [18]	37 years	2 years	Persecutory delusions, auditory hallucinations. Normal memory. Poor attention and concentration.	Gonadotropins, prolactin (onset soon after childbirth) TSH, ACTH (onset not defined)	

^∗^No diabetes insipidus in any of the patients. Growth hormone was not tested in any of the patients.

## Data Availability

No data were used to support the findings of this study.

## Abbreviations

ACTH: Adrenocorticotropic hormone
FSH: Follicle-stimulating hormone
IGF-1: Insulin-like growth factor-1
LH: Luteinizing hormone
MRI: Magnetic resonance imaging
TSH: Thyroid-stimulating hormone.
